# Intra and inter-session reliability of rapid Transcranial Magnetic Stimulation stimulus-response curves of tibialis anterior muscle in healthy older adults

**DOI:** 10.1371/journal.pone.0184828

**Published:** 2017-09-14

**Authors:** Elisabetta Peri, Emilia Ambrosini, Vera Maria Colombo, Mark van de Ruit, Michael J. Grey, Marco Monticone, Giorgio Ferriero, Alessandra Pedrocchi, Giancarlo Ferrigno, Simona Ferrante

**Affiliations:** 1 NearLab, Department of Electronics, Information and Bioengineering, Politecnico di Milano, Milano, Italy; 2 Department of Physical and Rehabilitative Medicine, Scientific Institute of Lissone IRCCS, Istituti Clinici Scientifici Maugeri, Lissone, MB, Italy; 3 Department of Biomechanical Engineering Faculty of Mechanical, Maritime and Materials Engineering, Delft University of Technology, Delft, The Netherlands; 4 Acquired Brain Injury Rehabilitation Alliance, School of Health Sciences, University of East Anglia, Norwich, United Kingdom; 5 Department of Medical Sciences and Public Health, University of Cagliari, Cagliari, Italy; University of Ottawa, CANADA

## Abstract

**Objective:**

The clinical use of Transcranial Magnetic Stimulation (TMS) as a technique to assess corticospinal excitability is limited by the time for data acquisition and the measurement variability. This study aimed at evaluating the reliability of Stimulus-Response (SR) curves acquired with a recently proposed rapid protocol on tibialis anterior muscle of healthy older adults.

**Methods:**

Twenty-four neurologically-intact adults (age:55–75 years) were recruited for this test-retest study. During each session, six SR curves, 3 at rest and 3 during isometric muscle contractions at 5% of maximum voluntary contraction (MVC), were acquired. Motor Evoked Potentials (MEPs) were normalized to the maximum peripherally evoked response; the coil position and orientation were monitored with an optical tracking system. Intra- and inter-session reliability of motor threshold (MT), area under the curve (AURC), MEP_max_, stimulation intensity at which the MEP is mid-way between MEP_max_ and MEP_min_ (I50), slope in I50, MEP latency, and silent period (SP) were assessed in terms of Standard Error of Measurement (SEM), relative SEM, Minimum Detectable Change (MDC), and Intraclass Correlation Coefficient (ICC).

**Results:**

The relative SEM was ≤10% for MT, I50, latency and SP both at rest and 5%MVC, while it ranged between 11% and 37% for AURC, MEP_max_, and slope. MDC values were overall quite large; e.g., MT required a change of 12%MSO at rest and 10%MSO at 5%MVC to be considered a real change. Inter-sessions ICC were >0.6 for all measures but slope at rest and MEP_max_ and latency at 5%MVC.

**Conclusions:**

Measures derived from SR curves acquired in <4 minutes are affected by similar measurement errors to those found with long-lasting protocols, suggesting that the rapid method is at least as reliable as the traditional methods. As specifically designed to include older adults, this study provides normative data for future studies involving older neurological patients (e.g. stroke survivors).

## Introduction

Neuroplasticity is an important marker for motor recovery during neurorehabilitation. One way to measure plasticity is by assessing corticospinal excitability (CSE) using Transcranial Magnetic Stimulation (TMS). TMS is a non-invasive, painless, and well-established technique to evaluate CSE in studies of motor learning and neurorehabilitation [1] [2]. When TMS is administered over the cortical motor area, a motor evoked potential (MEP) is extracted from the targeted muscle’s electromyogram [3]. The MEP has been the primary measure used to quantify CSE. One way to use the MEP is to draw the input-output relationship between stimulation intensity and the size of the MEP, i.e. the Stimulus-Response (SR) curve [4,5].

The SR curve provides comprehensive information of the excitability of the nervous system [5],[6]. SR curves are traditionally acquired delivering stimuli at predefined stimulation intensities, often between 90% and 150% of the resting motor threshold (MT), with an inter-stimulus interval (ISI) of >4s, and the acquisition time for a whole SR curve is typically >8 minutes [5,7,8], [9]. SR curves take into account the response of both neurons with lower threshold, which are in the directly stimulated core region, and those that are activated with higher threshold, either because they are intrinsically less excitable or because they are far from the site where the stimulus is delivered [3]. As a result, the SR curve allows the investigation of changes in excitability of different neuronal populations, in contrast to stimulation at a single intensity [10]. However, despite the limitations of stimulating at a single intensity this technique is still the most used method to explore excitability, using the mean of the responses as the outcome measure.

While assessing CSE changes in longitudinal studies [11–15], the reliability of TMS-related measures is of primary importance in order to assure that any observed change is above the trial-to-trial variability of the measure itself. A recent systematic review describing the reliability of TMS outcome measures of primary motor cortex excitability in healthy subjects concluded that the evidence base is insufficient and is negatively affected by problems with methodological design and statistical analysis [16]. Beaulieu et al. (2017) also pointed out the importance of reporting the Minimal Detectable Change (MDC) for generalization to future work. The MDC represents the minimum difference required to determine if a significant change has occurred in an individual. The lack of appropriate statistical assessment and a general misunderstanding of the concept of reliability have been underlined by Schambra et al. (2015), who propose guidelines for the rigorous testing of TMS outcome reliability [17]. They clarify the two main subtypes of reliability: the measurement error (or absolute reliability) which assesses the agreement between repeated measurements in an individual and is mainly used for longitudinal evaluative purposes, and the so called reliability_MP_ (or relative reliability; ‘MP’ standing for measurement property) which assesses how well an individual can be distinguished from the others and might be useful for diagnostic purposes.

Variability of TMS-related measures is due to both endogenous and exogenous sources [18], [19,20]. Spontaneous physiological fluctuation in excitability levels at both cortical and spinal level is a primary cause of endogenous variations on TMS-related measures [21]. Due to endogenous variability, reducing the length of the acquisition protocol is of utmost importance. Concerning exogenous variability, there are plenty of sources of which those crucial are: i) age and gender; ii) visual attention level [22]; iii) time of day the experiment is performed (related to cortisol levels) [23]; iv) the contraction level of the target muscle [19]; and v) the position and orientation of the coil over the target cortical area together with stimulation intensity [19,20], [24]. Whilst consensus is reached for the effect of most of these factors there are conflicting results regarding the effect of age. For example, Pitcher et al. (2003) showed that older adults required greater stimulus intensities to reach maximal motor output in the corticospinal projection to intrinsic hand muscles and were characterized by higher trial-to-trial variability with respect to young participants, especially at near threshold stimulation intensities [25]. However, a subsequent study did not observe aging-related changes in corticospinal stimulus-response curve characteristics in a population of exclusively male subjects [26]. Therefore, since no final conclusion about the effect of age on CSE can be found in the literature, there is the need of age-matched normative data to be used as a reference for changes in CSE in patients suffering neurological disease (e.g. stroke).

To minimise variability and allow quantifying CSE across different intensities it would be advantageous to acquire data for the SR curve rapidly. To shorten the acquisition time, Mathias and colleagues studied the minimum ISI to avoid inhibitory or facilitatory interactions between the responses of two consecutive stimuli [9]. They showed that the ISI can be reduced up to 1.4s without inducing a depression of CSE which is a well-known effect of 1Hz repetitive TMS [27]. They also observed that 60 stimuli are sufficient to construct a representative curve, thus demonstrating that reliable SR curves can be acquired in less than 2 minutes. Next to the possibility of reducing variability, the reduction of the acquisition time is crucial for transferring TMS-related measures from research to clinical practice. Indeed, not only it helps in reducing variability, but it can also increase the patient’s compliance, thus limiting dropout.

Whereas most studies explored MEP variability in upper limb and especially hand muscles, lower limb muscles are less commonly studied, e.g. the tibialis anterior (TA) or soleus muscle. Of the 34 studies selected in Beaulieu et al.’s 2017 systematic review [16], only 10 were focused on lower limb muscles. This may be caused by the generally moderate reliability found for MEPs in lower limb muscle of healthy participants [6,18,28] whilst in neurological patients (i.e. stroke, multiple sclerosis and incomplete spinal cord injury) even poorer results are obtained [29–31]. Nonetheless, the TA has a crucial role in the recovery of walking (i.e., to overcome the drop-foot phenomenon typical following e.g. stroke), and reliable information of CSE can be crucial for clinical decision making. Importantly, among all studies exploring reliability of the MEPs in lower limb muscles, only Cacchio and colleagues assessed reliability of the whole SR curves on healthy adults [6], whilst it allows investigation of excitability of different neuronal populations at the same time. The aim of this study is to investigate the inter- and intra-session reliability of measures derived from SR curves acquired rapidly from the TA muscle in healthy older adults, age-matched to stroke survivors. Both absolute and relative reliability, as well as MDC for TMS measures, are assessed.

## Materials and methods

### Participants and study design

Healthy community-dwelling participants with an age between 55 and 80 years and no previous history of neurological injury were recruited for the study. Exclusion criteria were any contraindication recommended for TMS [1]; presence of metal implants or cardiac pacemaker; history of epilepsy or migraine; neurological or systemic diseases; assumption of antidepressants and/or anxiolytics; and any lower extremity injuries in the three months prior to the first experimental session [32].

The subjects participated to two experimental sessions in a test-retest design. The two sessions were separated by 4–7 days, in agreement with textbooks on psychometric properties which recommend using different days, but no more than 2 weeks apart to assess reliability [16,33].

Participants were asked to get sufficient sleep (>6 h), avoid coffee and minimize alcohol consumption the day and night before the experiment. During each session, six SR curves were acquired on the TA muscle of the dominant leg, three at rest and three during isometric muscle contractions at 5% of the maximal voluntary contraction (MVC). The dominant leg was identified by asking the subject the preferred leg to kick a ball [34].

The research protocol was approved by the central ethical committee of Fondazione Salvatore Maugeri (number: 931 CE, date of approval: 10/03/2014) and conducted in accordance with the Declaration of Helsinki. All participants provided written informed consent to participate.

### Apparatus

#### EMG

Surface self-adhesive Ag/AgCl electrodes (Kendall^TM^, COVIDIEN) were placed in a bipolar configuration over the TA muscle. EMG signals were acquired by a multi-channel signal amplifier (Porti 32™, TMS International) and sampled at 2048 Hz.

#### TMS

A biphasic TMS stimulator (Magstim Rapid2, The Magstim Company, Dyfed, UK) with a double-cone coil was used to elicit MEPs. The coil position and orientation over the target cortical motor area were monitored in real-time using a frameless stereotaxic custom C++ software [35] interfacing with an optical tracking system (Polaris Vicra, Northern Digital Inc.). The software allowed to retrieve coil position and orientation between sessions and helped the operator maintaining the correct coil position and orientation within each session by providing feedback about any errors with respect to the predefined stimulation site and coil orientation. Furthermore, it prevented unnecessary stimuli when the coil was not properly placed over the hotspot. A Graphical User Interface (GUI) developed in Matlab was used to deliver the TMS stimuli, to online display the SR curve and to store the data [36].

#### Peripheral nerve stimulation

A current-controlled stimulator (RehaStim^TM^, HASOMED GmbH) was used to evoke the maximal evoked muscle response (Mmax).

#### Muscle force level

A load cell (Tekkal, Milan, Italy) was used to measure the force produced during the isometric muscle contractions of the TA muscle. The force was visually displayed to the participants to help them maintaining 5%MVC during active SR curves acquisition.

**Fig 1:**
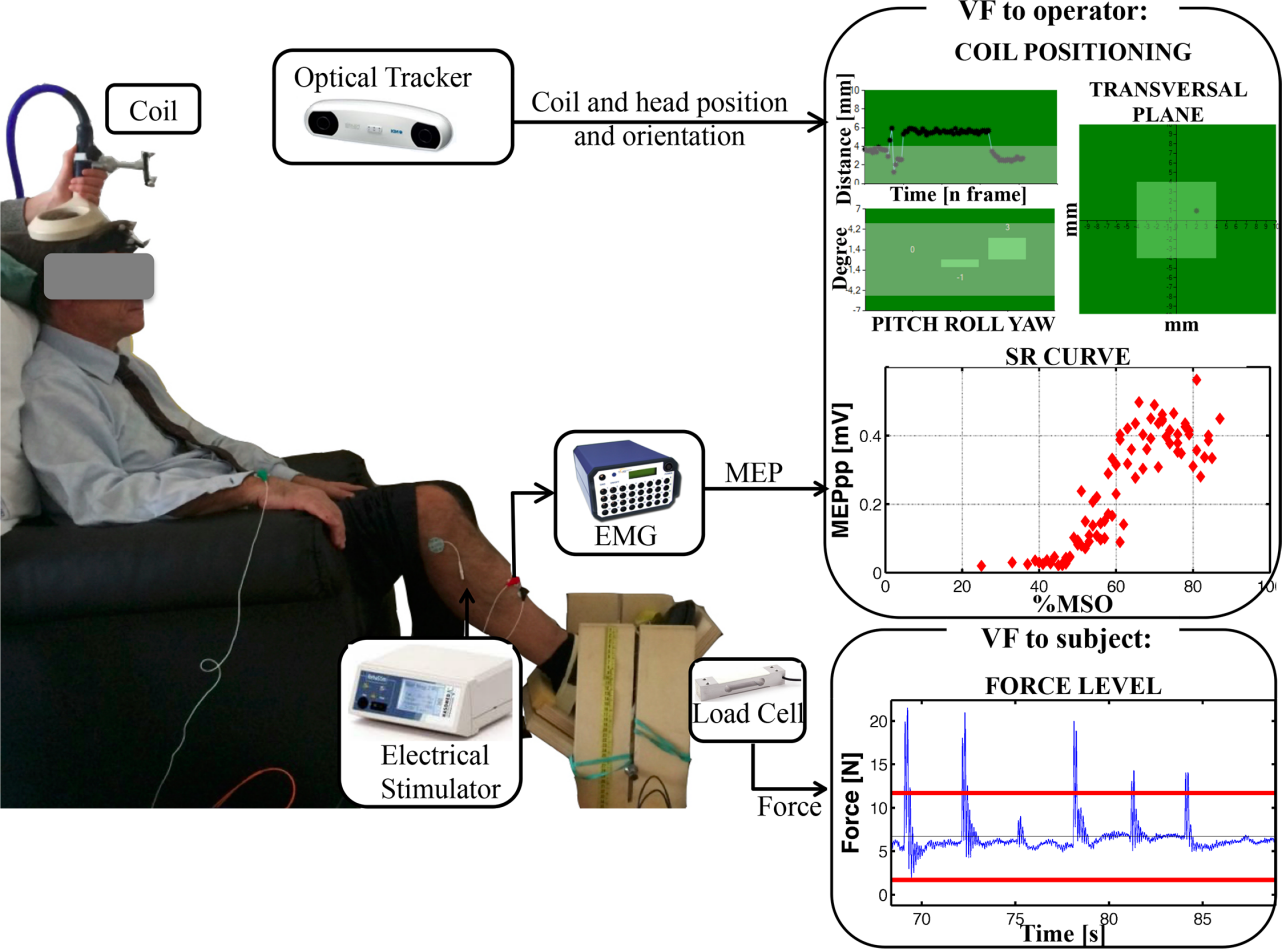
Experimental setup used for the acquisition of the SR curves. The coil position and orientation on the skull were monitored by an optical tracking system. An electrical stimulus was delivered to the peroneal nerve to elicit the maximum peripheral muscular response. The operators were provided with two visual feedbacks (VF): one GUI helped in maintaining the correct coil position and orientation (top right) and a second one visually displayed the SR curve while it was acquired (middle right). A load cell was used to monitor the force level produced during active SR curves, which was displayed to the participant (bottom right): the two red lines indicate the target range the participant was asked to maintain, the blue line shows the acquired force level.

The experimental setup is displayed in Fig 1.

### Experimental procedure

Participants were comfortably seated in a quiet room on an armchair with knee and ankle angles of the dominant leg fixed at about 100° and 90°, respectively. A custom-built wooden-made support maintained the correct position of the foot (see Fig 1).

At the beginning of each session supramaximal electrical stimuli were delivered to the peroneal nerve to evoke the Mmax. The recorded Mmax was used to normalize the MEPs collected on the same day in order reduce the test-retest variability due to electrodes replacement and to ensure a valid statistical comparison among participants [37,38]. Care was taken to consistently replace the electrodes between the two sessions.

During the first session, the optimal stimulation site (hotspot) was identified: the coil was moved in small steps over the TA cortical motor area in order to find the position and orientation which evoked the maximal MEPs in the TA muscle with the lowest stimulation intensity. Once found, the coil position and orientation were saved in the frameless stereotaxic software [35]. On the second day, the frameless stereotaxic software was used to reposition the coil with the same location and orientation with respect to the head as on the first day. With the coil firmly placed over the hotspot, six SR curves were collected using the rapid acquisition protocol described in [9], three with the TA muscle at rest and three during an isometric contraction at 5%MVC. For each SR curve, a train of stimuli was delivered with an ISI of 3s; the stimulation intensity varied pseudo-randomly on a pulse-by-pulse basis in an online adjustable range. The operator could adjust the minimum and maximum stimulation intensity accordingly to the online displayed SR curve in order to identify the threshold on one end and the plateau on the other end. The operator manually stopped the acquisition after 3–5 stimuli since the curve had reached a steady state (i.e., it did not change with successive stimuli). An ISI of 3s, intermediate with respect to those tested on upper limb muscles (1.4-4s) in [9], was selected in order to maximize the comfort of the participants. Indeed, higher stimulation intensities are required to evoke MEPs from the TA muscle. Furthermore, this value of ISI gave the subject enough time to recover the correct level of muscle contraction after the TMS stimulus during active SR curves acquisition.

As all participants were naïve TMS participants, at the beginning of the first session an additional short SR curve (30–40 stimuli) was acquired in order to familiarise the participants with the method. This curve was discarded from the analysis.

### Data analysis

The EMG signal was extracted from 150ms before to 300ms after each TMS stimulus and high-pass filtered (5^th^ order Butterworth filter, cut frequency of 5 Hz). The root mean square (RMS) of the EMG 100ms before the TMS stimulus, referred to as “background EMG”, was computed to monitor the state of the muscle before stimulation: individual MEPs were excluded from the subsequent analysis if their respective background EMG was over mean±3SD computed for the complete dataset of each SR curve.

The MEP size was computed as the peak-to-peak value (MEP_pp_) of the EMG signal in a 60ms window placed 20ms from the start of the TMS stimulus.

To construct the SR curve, MEP_pp_ values were first normalised to the peak-to-peak amplitude of the Mmax, then plotted as function of the stimulation intensity, and finally all data was modelled using a four-parameter Boltzmann sigmoid [9]:

$$MEP_{pp}(I)=MEP_{min}+\frac{MEP_{max}-MEP_{min}}{1+e^{\frac{I_{50}-I}{S}}}$$ (1)

where *MEP*_*min*_ and *MEP*_*max*_ are the minimum and maximum asymptotes of the function; I50 is the percentage of maximal stimulator output (%MSO) at which the MEP is mid-way between *MEP*_*min*_ and *MEP*_*max*_ and S is the slope at I50. The goodness of the fit was evaluated by means of the coefficient of determination R^2^. Curves with R^2^ ≤0.75 were discarded.

Corticospinal excitability was assessed in terms of:

1. Motor Threshold (MT), i.e. the minimum stimulus that evokes an MEP in the muscles and reflects the membrane excitability of the neurons in the cortical region of the target muscle [39]. It was computed as the x-intercept of the tangent to the sigmoid function at the point of maximal slope, i.e. I50 (see Equation I) [7]. It is expressed as %MSO.
2. Area Under Recruitment Curve (AURC), computed as the integral under the sigmoid function. This parameter provides a global estimate of the corticospinal excitability and is suggested to characterize the corticospinal projections to a wide range of muscles. An increase of the area indicates an increase of excitability [40].
3. MEP_max_, which reflects the maximum corticospinal response of the cortical neurons evoked by the stimulation [2].
4. I50, expressed as %MSO.
5. S, expressed as %MSO^-1^. It has been suggested to give inion about the neurophysiological strength of intracortical and corticospinal projections [39,41].
6. Latency, i.e. the time period between the TMS stimulus and the MEP onset. This parameter was computed only for MEPs elicited by stimuli with a stimulation intensity higher than the one corresponding to the 80% of the difference between maximum and minimum plateau of SR curve. Changes in MEP latency may reflects variation in central motor conduction time [6,42].
7. Silent period (SP), i.e. the period of EMG activity suppression following a supra-threshold TMS stimulus. It was computed only during muscle contractions and for the same MEPs selected to compute latency, as the time interval between the end of the MEP and the return of the background voluntary activity (i.e., >70% of the mean background EMG) [41]. SP is believed to reflect inhibitory mechanisms at the motor cortex level mediated by GABA-B receptors [42][41].

While MT, AURC, MEP_max_, I50, and slope were derived from the sigmoid function of the SR curve, latency and silent period were derived from individual MEPs.

### Statistical methods

An *a priori* power analysis showed that 22 was the minimum sample size required to establish that a reliability coefficient of 0.80 was significantly different from a minimally acceptable reliability coefficient of 0.50, considering *α* = 0.05 and 1-*β* = 0.80 [43]. A total of 24 participants were recruited to allow for a 10% drop-out rate.

After verifying the homoscedasticity of each dataset by means of the Breusch-Pagan test [44], the variance components of the observed measurements were estimated using the restricted maximum likelihood method and a random effects model, as follows [17]:

$$\sigma_{observed}^{2}=\sigma_{subjects}^{2}+\sigma_{tests\;or\;days}^{2}+\sigma_{residual}^{2}$$ (2)

where $\sigma_{subjects}^{2}$ is the between-subject variance, $\sigma_{tests\;or\;days}^{2}$ is the variance between the three measurements collected on the first day (intra-session) or the variance between days (inter-session), and $\sigma_{residual}^{2}$ is the error term which represents all other unexplained sources of variability. Please note that the variance components to derive inter-session reliability were estimated twice, once considering the average of the three daily measurements of each subject and once considering only the first measurement collected on each day.

The measurement error was estimated by the Standard Error of Measurement, which can be easily derived from the variance components as follows [17]:

$$SEM=\sqrt{\sigma_{tests\;or\;days}^{2}+\sigma_{residual}^{2}}$$ (3)

The SEM takes into account both systematic ($\sigma_{tests\;or\;days}^{2}$) and random error ($\sigma_{residual}^{2}$).

The relative SEM (SEM_rel_%) was also computed by normalizing the SEM to the measurement mean, as follows:

$${SEM}_{rel}\%=\frac{SEM}{mean}*100$$ (4)

From the SEM computed between sessions, the MDC, i.e. the smallest change in score that is likely to reflect a true change rather than a measurement error, was estimated as follows [45]:

$$MDC=SEM*1.96*\sqrt{2}$$ (5)

where $\sqrt{2}$ accounts for the variance associated with two independent sessions and 1.96 represents the 95% confidence interval.

Relative intra- and inter-session reliability of TMS-related measures was estimated by the Intraclass Correlation Coefficient (ICC), using the ICC(2,1) formula (model 2, random-effects 1-way, single measures) [17,45]. To take into account possible systematic differences, absolute agreement was selected. For intra-session reliability, the three measurements collected the first day were considered, while for inter-session reliability, as before, the average daily measurements and only the first measurement collected on each day were considered separately. ICC values >0.70 are usually interpreted as acceptable reliability_MP_ [17].

Repeated-measures ANOVA and paired t-tests were used to evaluate possible systematic errors between the three dataset collected on the first day (intra-session) and between the average daily measurements or only the first measurement collected on the two days (inter-session), respectively.

The measurement properties were computed separately for dataset acquired at rest and during muscle contractions (referred to as “active”).

Differences among the three dataset of the two sessions in terms of background EMG and force level were also investigated by means of repeated-measures ANOVA. A paired t-test was used to assess differences in terms of Mmax between the two sessions. Descriptive group data are reported as mean ± standard deviation unless otherwise noted.

The statistical analysis was performed with IBM SPSS Statistics v23 software.

## Results

Twenty-four healthy participants (12 males and 12 females) aged between 55 and 75 years old were recruited. Participants’ details are provided in Table 1.

**Table 1:** Participants' details.

N	24
Age * [years]	62.3 ± 4.5
Height * [cm]	168 ± 9
Gender (M/F)	12/12
Dominant leg (R/L)	21/3

* indicates mean±SD. M (male), F (female), R (right), L (left)

Two participants (age 64 and 69 years) did not return for the second session, while the MT at rest was >100%MSO for one subject (64 years) and therefore MEPs at rest could not be evoked. Thus, the intra-session reliability analysis was based on 23 and 24 subjects at rest and 5%MVC, respectively, while for the inter-session reliability analysis 21 and 22 subjects were considered for the passive and active conditions, respectively.

Each SR curve was acquired by delivering an average of 70±2 stimuli at rest and 67±3 during muscle contractions. Thus, the overall duration of each SR curve acquisition was of about 3.5 minutes. None or one MEP (average of 0.09%±0.11%) were excluded because of the background EMG. The SR curves obtained a coefficient of determination R^2^ = 0.85±0.08, with values always bigger than 0.75.

The Mmax was not significantly different between the two sessions (5.3±2.0 at day 1 and 5.8±2.6 at day 2, p = 0.388).

The participants were able to maintain the TA contractions at 5%MVC as required, exploiting the visual feedback of the force level. Indeed, no significant differences were obtained for the six active SR curves neither in terms of force level (F = 0.75, p = 0.595) nor in terms of background EMG (F = 0.55, p = 0.735).

**Fig 2:**
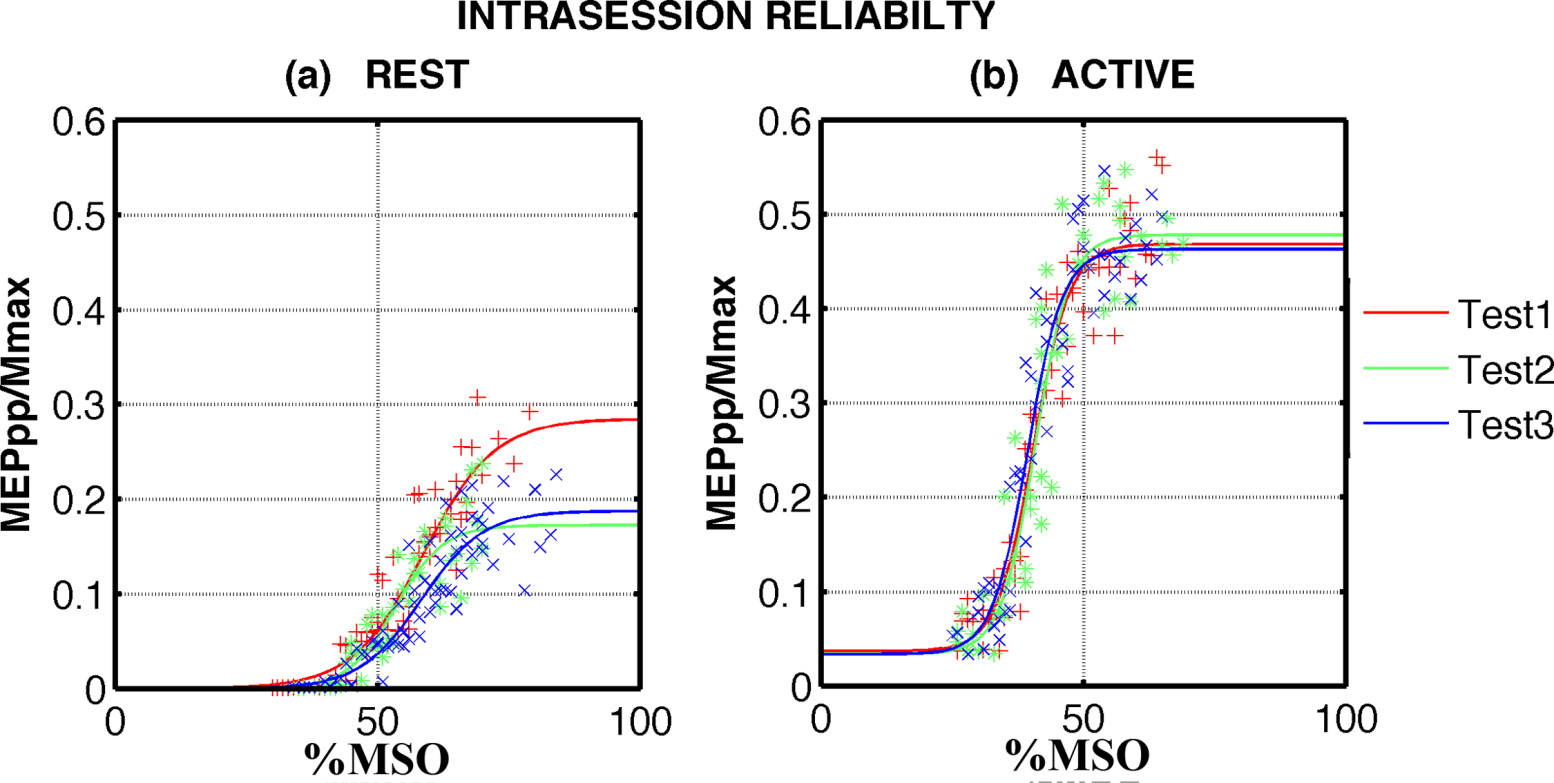
Exemplary dataset acquired within one session for one participant. Raw data and sigmoid functions obtained on the first day at rest (panel (a)) and at 5%MVC (panel (b)) are shown as red, green and blue dots and corresponding lines.

**Fig 3:**
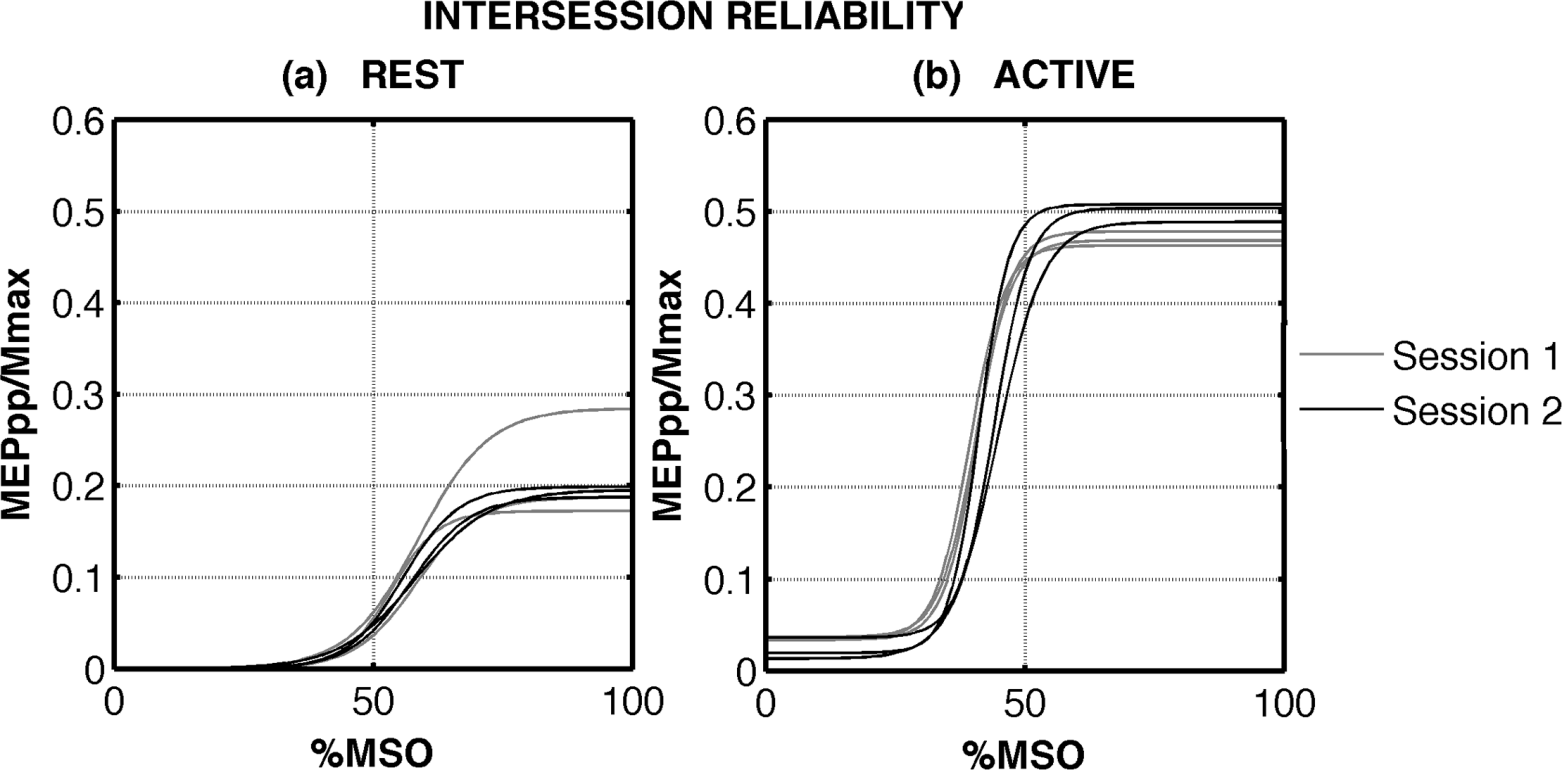
Exemplary dataset acquired in the two sessions for one participant. The three fitted SR curves obtained during session 1 (in grey) and session 2 (in black) at rest (panel (a)) and at 5%MVC (panel (b)) are shown.

Exemplary dataset acquired on one single subject (female, 63 years old, right dominant leg) are shown in Figs 2 and 3. Active SR curves (panels (b)) show lower motor thresholds, steeper slopes, higher MEP_max_, and higher AURC compared to the passive (panels (a)).

All dataset was found to be homoscedastic and therefore no transformation was needed to compute the measurement properties.

### Intra-session reliability

The results of the intra-session reliability analysis are reported in Table 2. Systematic errors were found only for the silent period (repeated measures ANOVA, p<0.01). All the ICCs were significant (p-value<0.001). The measurement error for MT was <4%MSO both at rest and at 5%MVC. Analogously, the measurement error for MEP_max_, slope, and latency were similar between rest and active conditions and about 0.08, 2%MSO^-1^, and 2ms, respectively. For the AURC, the SEM increased from 1.7 at rest to 3.3 during muscle contractions. Opposite was the behaviour of I50, whose SEM decreased from 5.5%MSO at rest to 3.8%MSO at 5%MVC. The relative SEM was always below or about 10% except for the AURC at rest (16%) and the MEP_max_ and the slope in both conditions (MEP_max_: 26% and 12% at rest and at 5%MVC; slope: 37% and 29%). A relative SEM <10% was previously proposed as a cut-off for high measurement stability [17,46]. Considering this cut-off, all measurements but AURC at rest and MEP_max_ and slope in both conditions were characterized by a low relative measurement error within the same session. ICC values were >0.70 for all parameters but slope at rest (ICC = 0.54) and SP (ICC = 0.67), suggesting a good reliability_MP_, which is the ability of the measurement to distinguish between subjects in a sample.

**Table 2:** Intra-session reliability analysis of the TMS-related parameters obtained at rest and at 5%MVC (active).

		Test 1*			Test 2*			Test 3*			P-value^†^	SEM	SEM_rel_ (%)	ICC (95% CI)
**Rest (N = 23)**	**MT [%MSO]**	58	±	13	57	±	13	58	±	14	0.68	3.7	6.4	0.92 (0.85; 0.96)
	**AURC**	11	±	10	10	±	9	10	±	8	0.49	1.7	16.5	0.96 (0.93; 0.98)
	**MEP**_**max**_	0.31	±	0.21	0.29	±	0.21	0.28	±	0.17	0.42	0.08	25.7	0.85 (0.73; 0.93)
	**I50[%MSO]**	69	±	16	69	±	14	69	±	16	0.97	5.5	8.0	0.87 (0.76; 0.94)
	**Slope[%MSO]**^**-1**^	5.4	±	3.0	5.8	±	2.9	5.5	±	3.1	0.64	2.0	36.7	0.54 (0.29; 0.75)
	**Latency [ms]**	24	±	5	24	±	5	24	±	4	0.49	1.8	7.5	0.82 (0.68; 0.92)
**Active (N = 24)**	**MT [%MSO]**	42	±	8	42	±	9	41	±	10	0.85	3.9	9.4	0.82 (0.68; 0.91)
	**AURC**	30	±	11	29	±	11	31	±	12	0.36	3.3	11.0	0.92 (0.85; 0.96)
	**MEP**_**max**_	0.65	±	0.22	0.6	±	0.17	0.64	±	0.2	0.09	0.08	12.3	0.84 (0.71; 0.92)
	**I50 [%MSO]**	57	±	15	55	±	13	56	±	12	0.21	3.8	6.7	0.92 (0.85; 0.96)
	**Slope [%MSO]**^**-1**^	6.6	±	4.7	5.5	±	3.4	5.7	±	3.2	0.07	1.7	29.2	0.80 (0.65; 0.90)
	**Latency [ms]**	29	±	5	29	±	6	30	±	4	0.25	2.1	7.2	0.82 (0.67; 0.91)
	**SP [ms]**	140	±	26	153	±	26	158	±	28	<0.01	16.1	10.7	0.67 (0.39; 0.84)

* mean ± SD obtained across participants during the 3 repetition of the first day (Test 1, Test 2 and Test 3)

† Repeated measures ANOVA

MT: motor threshold; AURC: area under the recruitment curve; SP: silent period

MSO: maximal stimulator output

SEM: Standard Error of Measurement; SEM_rel_%: relative Standard Error of Measurement; ICC (CI 95%): intraclass correlation coefficient (95% confidence interval)

### Inter-session reliability

The results of the inter-session reliability analysis based on the mean values of the 3 curves acquired during each testing session are shown in Table 3. The ICC of the slope at rest was not significant (p-value = 0.121); all the others were significant (p-value<0.02). The measurement errors for AURC and MEP_max_ were higher than those obtained within a single session. These measures, as well as the slope, showed a relative SEM >10%, indicating a low measurement stability. All the other measures exhibited a relative SEM about or below 10%. The MDC values were overall quite large; for example, MT required a change of at least 12%MSO at rest and 10%MSO at 5%MVC to be considered a real change above the measurement error. A good reliability_MP_ was found for MT and I50, both at rest and during muscle contractions, AURC and MEP_max_ at rest, slope during muscle contractions, and SP, with ICC values >0.70.

**Table 3:** Inter-session reliability analysis of TMS-related parameters at rest and at 5%MVC (active).

		Test*			Retest*			P-value^†^	SEM	SEM_rel_ (%)	MDC	ICC (95% CI)
**Rest (N = 21)**	**MT [%MSO]**	56	±	12	55	±	9	0.40	4.4	7.9	12.2	0.84 (0.64; 0.93)
	**AURC**	11	±	9	9	±	8	0.04	3	30.0	8.3	0.88 (0.70; 0.95)
	**MEP**_**max**_	0.30	±	0.19	0.23	±	0.14	0.02	0.09	35.8	0.26	0.71 (0.37; 0.87)
	**I50 [%MSO]**	67	±	14	68	±	14	0.89	7.4	11.0	20.5	0.72 (0.43; 0.88)
	**Slope [%MSO]**^**-1**^	5.7	±	2.4	5.7	±	2.6	0.97	2.1	37.1	5.8	0.28 (-0.20; 0.64)
	**Latency [ms]**	23	±	4	24	±	5	0.24	2.5	10.6	6.9	0.66 (0.33; 0.84)
**Active (N = 22)**	**MT [%MSO]**	42	±	9	41	±	7	0.45	3.7	8.9	10.3	0.77 (0.52; 0.90)
	**AURC**	31	±	12	30	±	14	0.93	8	26.2	22.2	0.60 (0.24; 0.81)
	**MEP**_**max**_	0.62	±	0.19	0.59	±	0.26	0.54	0.17	28.1	0.47	0.44 (0.02; 0.72)
	**I50 [%MSO]**	55	±	13	54	±	12	0.05	3.2	5.9	9.0	0.94 (0.84; 0.97)
	**Slope [%MSO]**^**-1**^	5.5	±	3.4	5.2	±	3.9	0.38	1.3	25.2	3.7	0.87 (0.71; 0.94)
	**Latency [ms]**	29	±	4	29	±	4	0.55	3	10.3	8.3	0.54 (0.16; 0.78)
	**SP [ms]**	155	±	27	148	±	23	0.20	13.8	9.1	38.3	0.71 (0.42; 0.87)

* mean ± SD obtained across participants at day 1 (Test) and day 2 (Retest); group means were obtained by first averaging each subject’s daily measurements.

† Paired t-test

MT: motor threshold; AURC: area under the recruitment curve; SP: silent period

MSO: maximal stimulator output

SEM: Standard Error of Measurement; SEM_rel_%: relative Standard Error of Measurement; MDC: Minimum Detectable Changes; ICC (CI 95%): intraclass correlation coefficient (95% confidence interval)

To evaluate whether it was possible to further reduce the acquisition time, the inter-session reliability analysis was performed also just considering the first curve acquired at rest and at 5%MVC during the two sessions. Results are reported in Table 4. All the ICCs were significant (p-value<0.03), with the only exception of the slope at rest (p-value = 0.671). Both reliability measurement properties at rest were overall worsened and in the majority of the cases did not reached acceptable values. MT and I50 at 5% MVC maintained a relative SEM <10%, indicating a good measurement stability, and ICC values >0.7.

**Table 4:** Inter-session reliability analysis of TMS-related parameters at rest and at 5%MVC (active). Only the first curves acquired in the two sessions are considered.

		Test*			Retest*			P-value^†^	SEM	SEM_rel_ (%)	MDC	ICC (95% CI)
**Rest (N = 21)**	**MT [%MSO]**	57	±	13	53	±	9	0.16	7.4	13.5	20.5	0.54 (0.17; 0.78)
	**AURC**	11	±	10	9	±	8	0.12	3.8	38.0	10.5	0.82 (0.61; 0.92)
	**MEP**_**max**_	0.32	±	0.22	0.23	±	0.14	0.02	0.11	41.5	0.32	0.63 (0.26; 0.84)
	**I50 [%MSO]**	67	±	15	67	±	15	0.98	9.67	14.4	26.8	0.57 (0.19; 0.80)
	**Slope [%MSO]**^**-1**^	5.4	±	3.0	5.6	±	3.3	0.74	3.2	57.8	8.8	-0.11 (-0.55; 0.36)
	**Latency [ms]**	24	±	4	24	±	6	0.75	2.8	11.7	7.8	0.68 (0.36; 0.86)
**Active (N = 22)**	**MT [%MSO]**	42	±	8	41	±	8	0.88	3.2	7.7	8.8	0.77 (0.51; 0.90)
	**AURC**	30	±	12	30	±	14	0.93	8	26.7	22.2	0.60 (0.24; 0.81)
	**MEP**_**max**_	0.63	±	0.22	0.58	±	0.24	0.32	0.18	29.6	0.50	0.40 (-0.00; 0.70)
	**I50 [%MSO]**	56	±	15	53	±	12	0.02	5.0	9.2	13.9	0.86 (0.65; 0.95)
	**Slope [%MSO]**^**-1**^	6.1	±	4.6	5.1	±	3.8	0.02	1.6	29.1	4.5	0.85 (0.64; 0.94)
	**Latency [ms]**	28	±	5	29	±	5	0.73	3.4	11.9	9.4	0.49 (0.09; 0.75)
	**SP [ms]**	151	±	32	139	±	27	0.06	19.9	13.7	55.2	0.57 (0.20; 0.80)

* mean ± SD obtained across participants during the first repetition of day 1 (Test) and day 2 (Retest).

† Paired t-test

MT: motor threshold; AURC: area under the recruitment curve; SP: silent period

MSO: maximal stimulator output

SEM: Standard Error of Measurement; SEM_rel_%: relative Standard Error of Measurement; MDC: Minimum Detectable Changes; ICC (CI 95%): intraclass correlation coefficient (95% confidence interval)

## Discussion

This study assessed the reliability of TMS-related measures collected from the TA muscle at rest and at 5%MVC of a population of 24 healthy older adults (mean age of 62 years). These measures, although acquired with the rapid protocol for SR curves proposed in [9], were similar, in terms of magnitude, to those previously obtained on the TA muscle of healthy subjects [6,18,29,30,47]. Comparisons were not possible for the AURC and I50, since no previous studies evaluated this outcome on the TA muscle. The measurement error between sessions were overall quite large. However, all measures but AURC, MEP_max_, and slope could be considered as acceptable, with a relative measurement error ≤10% [17].

The lowest relative measurement errors were found for MT and I50 both at rest and at 5%MVC. MT was characterized by MDC values of 12.2%MSO (at rest) and 10.2%MSO (at 5%MVC), comparable to 9.3%MSO for resting MT found in [6,29]. Therefore, we concluded that the MT is reliable even when derived from the SR curve instead of using the traditional method (MT is usually defined as the lowest stimulation intensity inducing a MEPpp >50μV in at least 5 out of 10 consecutive trials [6,18,29]). Comparisons were not possible for I50 since no previous data were found.

MEP_max_ showed a measurement error above the cut-off of 10%, as already observed in [6]: we obtained a value of SEM_rel_ equal to 36% at rest and 28% at 5%MVC, compared to 16% found at rest in [6]. The slope showed a higher measurement error with respect to what previously found: in [6] the Authors observed a relative SEM of 8.2% for the TA at rest, while we found a relative SEM of 25.1% and 37.1% at rest and at 5%MVC, respectively.

Instead of considering each single curve parameter individually, AURC has been candidate as a global indicator of cortical excitability [40] and therefore as the most clinically meaningful outcome to be used in longitudinal studies. Our study assessed for the first time its measurement properties for lower limb muscles: high values of SEM_rel_ were found between sessions (30% at rest and 26% at 5%MVC), indicating that big changes are needed to overcome the measurement error. This high measurement error reflected the high errors already found for MEP_max_ and slope.

When compared to the literature, higher and lower MDC values were found for latency (7/8ms versus <2ms found in [6,29]), and SP (38ms versus 60ms in [6]), respectively. However, comparisons must be made with caution since previous studies have assessed these outcomes at different stimulation intensities and the stimulation intensity has been seen to influence the magnitude of these measures: SP increases and latency decreases with increased stimulation intensity [48,49]. Furthermore, previous studies acquired active SR curves at contraction levels higher than 5%MVC (20%MVC in [6] and 10%MVC in [29]). Although this low background activity made comparison with the literature more difficult, it was chosen to reduce the risk of muscle fatigue, particularly relevant for healthy older adults and, even more, for neurological patients (e.g. stroke survivors).

Overall, we observed that individual changes needed to exceed the MDC values should be quite high; for example, we obtained that MT during slight muscle contractions should change of >10%MSO after an intervention to be considered a real change above the measurement error (MDC = 10.3%MSO at 5% MVC, as reported in Table 3). However, such a change is quite unusual to be observed [17]. Therefore, as already suggested in [17] and confirmed in [16], MDC values should be better used to identify changes within a homogenous group of subjects rather than to track individual changes. Indeed, in case of groups, MDC value is divided by the square root of the sample size and its value is strongly reduced, even for small samples.

Compared to absolute reliability, relative reliability was more commonly investigated in the literature. Several studies have estimated ICC values for TMS outcomes in TA muscle on healthy subjects [6,18,29,30,47]. Good relative reliability (ICC>0.70) was generally found for resting MT, slope, latency, silent period, and MEP amplitude; only one study [30] observed a moderate reliability for latency (ICC of 0.55–0.71 during isometric muscle contractions of 10% to 60%MVC) and silent period (ICC of 0.16–0.40). Similar results were observed in our study for MT (inter-session ICC of 0.84 and 0.77 at rest and at 5%MVC, respectively) and SP (ICC of 0.71), while a moderate reliability as in [30] was obtained for latency (ICC of 0.66 and 0.54 at rest and at 5%MVC, respectively). A lower reliability with respect to the literature was found for the slope at rest (inter-session ICC of 0.28 versus 0.78 found in [6]); however, during slight muscular contraction a good relative reliability was regained (ICC of 0.87). For MEP_max_, the same value of ICC was found in our study and in [6] at rest (ICC of 0.71). Concerning the AURC, ICC ranged from 0.88 at rest to 0.60 at 5%MVC, and to our knowledge no other studies evaluated its reliability_MP_ so far. Based on our results, one could conclude that all TMS measures but AURC and MEP_max_ during muscle contraction, latency, and slope at rest could be used to discriminate between subjects for staging or diagnosis.

As already observed [6,16], lower measurement errors were found when outcomes were acquired within the same day (intra-session, Table 2) than some days apart (inter-session, Table 3), in particular for AURC and MEP_max_. This is mainly due to the different sources of variability which affect intra- and inter-session reliability. The measurement error within the same day is mainly due to the physiological fluctuations of the excitability at cortical and spinal levels. Reducing the acquisition time to 3–4 minutes we expected to decrease the effect of long-term exogenous variability, and so to reduce the intra-session variability. However, this result was not achieved. The higher measurement error between different sessions is most likely due to the methodological sources of variability, such as EMG electrodes replacement and hotspot repositioning [16]. The use of an optical electronic system and a custom-made software to maintain the same coil position and orientation within and between sessions did not reduce the measurement variability, as already observed [50].

As expected, averaging the parameters over 3 curves increases the reliability with respect to single-curve parameters, since the average reduces the effect of random errors. Therefore, when studying small changes in corticospinal excitability, usually found in healthy participants following brief motor learning paradigms, three SR curves may be needed to optimally quantify changes in excitability. However, in patients where changes in CSE are expected to be large, a single curve will be sufficient to detect changes.

Our study has some limitations. Firstly, an ISI<3s could have been investigated in order to further reduce the acquisition time of the SR curves. Secondly, the study did not collect active SR curves at contraction levels >5%MVC (e.g. 10–20%MVC) and this limited the possibility to compare our results with previous findings.

## Conclusion

This study showed that although a shorter time for data collection and an experimental protocol designed to minimise measurement variability, TMS measures acquired by stimulating the area of the motor cortex representing the TA muscle of healthy older adults using this method are comparable to traditional methods and are affected by a large measurement error. Therefore, our results support the use of TMS measures to detect changes significantly over the measurement error in group of subjects, instead of individual changes. In such a way, when used in longitudinal studies aimed at investigating neuroplasticity linked to motor rehabilitation, TMS measures might aid our understanding about how we can augment the effect of motor rehabilitation and identify the optimal treatment plans for its effects to persist and translate to improvements in daily life activities. As specifically designed to include older adults, this study provides normative data for future studies involving older neurological patients (e.g. stroke survivors).

## Supporting information

S1 File MT, AURC, slope, I50, MEP_max_, latency, SP computed for each of the 24 patients are reported for passive (sheet 1) and active (sheet 2) test conditions. Each column corresponds to data extracted from a single SR curve (three at Day1 and three at Day2).
(XLSX)

## References

[1] Rossi S, Hallett M, Rossini PM, Pascual-Leone A. Safety, ethical considerations, and application guidelines for the use of transcranial magnetic stimulation in clinical practice and research. Clin Neurophysiol. 2012;120: 323–330. doi: 10.1016/j.clinph.2009.08.016.Rossi 10.1016/j.clinph.2009.08.016 PMC3260536 19833552

[2] Rothwell John C. Transcranial electrical and magnetic stimulation of the brain: basic physiological mechanisms In: Hallett M, Chokroverty S eds, editor. Magnetic Stimulation in Clinical Neurophysiology. Elsevier; 2005 pp. 43–60.

[3] Hallett M. Transcranial magnetic stimulation and the human brain. Nature. Macmillian Magazines Ltd.; 2000;406: 147–50. doi: 10.1038/35018000 10910346 10.1038/35018000

[4] Ridding MC, Rothwell JC. Stimulus/response curves as a method of measuring motor cortical excitability in man. Electroencephalogr Clin Neurophysiol Mot Control. 1997;105: 340–344. doi: 10.1016/S0924-980X(97)00041-6 10.1016/s0924-980x(97)00041-6 9362997

[5] Devanne H, Lavoie BA, Capaday C. Input-output properties and gain changes in the human corticospinal pathway. Exp Brain Res. 1997;114: 329–338. doi: 10.1007/PL00005641 9166922 10.1007/pl00005641

[6] Cacchio A, Cimini N, Alosi P, Santilli V, Marrelli A. Reliability of transcranial magnetic stimulation-related measurements of tibialis anterior muscle in healthy subjects. Clin Neurophysiol. 2009;120: 414–9. doi: 10.1016/j.clinph.2008.11.019 19135412 10.1016/j.clinph.2008.11.019

[7] Carroll TJ, Riek S, Carson RG. Reliability of the input–output properties of the cortico-spinal pathway obtained from transcranial magnetic and electrical stimulation. J Neurosci Methods. 2001;112: 193–202. doi: 10.1016/S0165-0270(01)00468-X 11716954 10.1016/s0165-0270(01)00468-x

[8] Everaert DG, Thompson AK, Chong SL, Stein RB. Does functional electrical stimulation for foot drop strengthen corticospinal connections? Neurorehabil Neural Repair. SAGE Publications; 2010;24: 168–77. doi: 10.1177/1545968309349939 19861590 10.1177/1545968309349939

[9] Mathias JP, Barsi GI, Van De Ruit M, Grey MJ. Rapid acquisition of the transcranial magnetic stimulation stimulus response curve. Brain Stimul. Elsevier Ltd; 2014;7: 59–65. doi: 10.1016/j.brs.2013.08.003 24120355 10.1016/j.brs.2013.08.003

[10] Di Lazzaro V, Profice P, Ranieri F, Capone F, Dileone M, Oliviero A, et al I-wave origin and modulation. Brain Stimul. 2012;5: 512–525. doi: 10.1016/j.brs.2011.07.008 21962980 10.1016/j.brs.2011.07.008

[11] Cortes M, Black-Schaffer RM, Edwards DJ. Transcranial Magnetic Stimulation as an Investigative Tool for Motor Dysfunction and Recovery in Stroke: An Overview for Neurorehabilitation Clinicians. Neuromodulation Technol Neural Interface. Blackwell Publishing Inc; 2012;15: 316–325. doi: 10.1111/j.1525-1403.2012.00459.x 22624621 10.1111/j.1525-1403.2012.00459.x PMC3760962

[12] Swayne OBC, Rothwell JC, Ward NS, Greenwood RJ. Stages of motor output reorganization after hemispheric stroke suggested by longitudinal studies of cortical physiology. Cereb Cortex. Oxford University Press; 2008;18: 1909–22. doi: 10.1093/cercor/bhm218 18234688 10.1093/cercor/bhm218 PMC2474452

[13] Takechi U, Matsunaga K, Nakanishi R, Yamanaga H, Murayama N, Mafune K, et al Longitudinal changes of motor cortical excitability and transcallosal inhibition after subcortical stroke. Clin Neurophysiol. 2014;125: 2055–2069. doi: 10.1016/j.clinph.2014.01.034 24636830 10.1016/j.clinph.2014.01.034

[14] Xu Y, Hou Q-H, Russell SD, Bennett BC, Sellers AJ, Lin Q, et al Neuroplasticity in post-stroke gait recovery and noninvasive brain stimulation. Neural Regen Res. Medknow Publications; 2015;10: 2072–80. doi: 10.4103/1673-5374.172329 26889202 10.4103/1673-5374.172329 PMC4730838

[15] Rossini PM, Ferilli MAN, Rossini L, Ferreri F. Clinical neurophysiology of brain plasticity in aging brain. Curr Pharm Des. 2013;19: 6426–39. 23432716 10.2174/1381612811319360004

[16] Beaulieu L-D, Flamand VH, Massé-Alarie H, Schneider C. Reliability and minimal detectable change of transcranial magnetic stimulation outcomes in healthy adults: A systematic review. Brain Stimul. 2017;10: 196–213. doi: 10.1016/j.brs.2016.12.008 28031148 10.1016/j.brs.2016.12.008

[17] Schambra HM, Ogden RT, Martínez-Hernández IE, Lin X, Chang YB, Rahman A, et al The reliability of repeated TMS measures in older adults and in patients with subacute and chronic stroke. Front Cell Neurosci. 2015;9: 335 doi: 10.3389/fncel.2015.00335 26388729 10.3389/fncel.2015.00335 PMC4555014

[18] Tallent J, Goodall S, Hortobogyi T, St Clair Gibson A, French DN, Howatson G. Repeatability of corticospinal and spinal measures during lengthening and shortening contractions in the human tibialis anterior muscle. PLoS One. 2012;7: 1–8. doi: 10.1371/journal.pone.0035930 22563418 10.1371/journal.pone.0035930 PMC3338551

[19] Kiers L, Cros D, Chiappa KH, Fang J. Variability of motor potentials evoked by transcranial magnetic stimulation. Electroencephalogr Clin Neurophysiol. 1993;89: 415–23. 7507428 10.1016/0168-5597(93)90115-6

[20] Schmidt S, Bathe-Peters R, Fleischmann R, Rönnefarth M, Scholz M, Brandt SA. Nonphysiological factors in navigated TMS studies; confounding covariates and valid intracortical estimates. Hum Brain Mapp. 2015;36: 40–9. doi: 10.1002/hbm.22611 25168635 10.1002/hbm.22611 PMC6869746

[21] Adrian ED, Moruzzi G. Impulses in the pyramidal tract. J Physiol. 1939;97: 153–99. 16995153 10.1113/jphysiol.1939.sp003798 PMC1393899

[22] Kamke MR, Hall MG, Lye HF, Sale M V, Fenlon LR, Carroll TJ, et al Visual attentional load influences plasticity in the human motor cortex. J Neurosci. 2012;32: 7001–8. doi: 10.1523/JNEUROSCI.1028-12.2012 22593068 10.1523/JNEUROSCI.1028-12.2012 PMC6622206

[23] Sale M V, Ridding MC, Nordstrom MA. Cortisol inhibits neuroplasticity induction in human motor cortex. J Neurosci. 2008;28: 8285–93. doi: 10.1523/JNEUROSCI.1963-08.2008 18701691 10.1523/JNEUROSCI.1963-08.2008 PMC6670557

[24] Ridding MC, Ziemann U. Determinants of the induction of cortical plasticity by non-invasive brain stimulation in healthy subjects. J Physiol. 2010;588: 2291–2304. doi: 10.1113/jphysiol.2010.190314 20478978 10.1113/jphysiol.2010.190314 PMC2915507

[25] Pitcher JB, Ogston KM, Miles TS. Age and sex differences in human motor cortex input-output characteristics. J Physiol. Blackwell Publishing Ltd; 2003;546: 605–613. doi: 10.1113/jphysiol.2002.029454 12527746 10.1113/jphysiol.2002.029454 PMC2342521

[26] Smith AE, Sale M V, Higgins RD, Wittert G a, Pitcher JB. Male human motor cortex stimulus-response characteristics are not altered by aging. J Appl Physiol. 2011;110: 206–12. doi: 10.1152/japplphysiol.00403.2010 21071590 10.1152/japplphysiol.00403.2010

[27] Chen R. Depression of motor cortex excitability by low-frequency transcranial magnetic stimulation. Neurology. 1997;48: 1398–1403. 9153480 10.1212/wnl.48.5.1398

[28] Peri E, Colombo VM, Ambrosini E, van de Ruit M, Grey MJ, Monticone M, et al Reliability of Rapid TMS Stimulus-Response Curves During Tibialis Anterior Contractions on Healthy Elderly. Springer International Publishing; 2016 pp. 1069–1074. doi: 10.1007/978-3-319-32703-7_211

[29] Cacchio A, Paoloni M, Cimini N, Mangone M, Liris G, Aloisi P, et al Reliability of TMS-related measures of tibialis anterior muscle in patients with chronic stroke and healthy subjects. J Neurol Sci. 2011;303: 90–94. doi: 10.1016/j.jns.2011.01.004 21262510 10.1016/j.jns.2011.01.004

[30] Van Hedel HJA, Murer C, Dietz V, Curt A. The amplitude of lower leg motor evoked potentials is a reliable measure when controlled for torque and motor task. J Neurol. 2007;254: 1089–1098. doi: 10.1007/s00415-006-0493-4 17431701 10.1007/s00415-006-0493-4

[31] Meaney A, Collett J, Dawes H, Howells K, Izadi H. Consistency of evoked responses to dual-stimulator, single-pulse transcranial magnetic stimulation in the lower limb of people with multiple sclerosis. J Clin Neurosci. Elsevier; 2015;22: 1434–1437. doi: 10.1016/j.jocn.2015.02.034 26154149 10.1016/j.jocn.2015.02.034

[32] Keel JC, Smith MJ, Wassermann EM. A safety screening questionnaire for transcranial magnetic stimulation. Clin Neurophysiol. 2001;112: 720 11332408 10.1016/s1388-2457(00)00518-6

[33] Portney LG, Watkins MP. Foundations of Clinical Research: Applications to Practice. Third ed. Upper Saddle River, editor. Pearson Education, Inc; 2009.

[34] Chapman JP, Chapman LJ, Allen JJ. The measurement of foot preference. Neuropsychologia. 1987;25: 579–584. doi: 10.1016/0028-3932(87)90082-0 3683814 10.1016/0028-3932(87)90082-0

[35] Ambrosini E, Biguzzi S, van de Ruit M, Pedrocchi A, Ferrigno G, Ferrante S, et al. Open-Source Software for Manual Transcranial Magnetic Stimulation Coil Positioning. 7th International IEEE EMBS Conference on Neural Engineering, Montpellier, France. 2015.

[36] van de Ruit M, Ambrosini E, Ferrante S, Grey MJ. Towards use of TMS as a clinical tool to assess plasticity. 26th Annual Meeting of Scoiety for the Neural Control of Movement.

[37] Groppa S, Oliviero A, Eisen A, Quartarone A, Cohen LG, Mall V, et al A practical guide to diagnostic transcranial magnetic stimulation: Report of an IFCN committee. Clin Neurophysiol. International Federation of Clinical Neurophysiology; 2012;123: 858–882. doi: 10.1016/j.clinph.2012.01.010 22349304 10.1016/j.clinph.2012.01.010 PMC4890546

[38] Rossini PM, Burke D, Chen R, Cohen LG, Daskalakis Z, Di Iorio R, et al Non-invasive electrical and magnetic stimulation of the brain, spinal cord, roots and peripheral nerves: Basic principles and procedures for routine clinical and research application. An updated report from an I.F.C.N. Committee. Clin Neurophysiol. 2015;126: 1071–107. doi: 10.1016/j.clinph.2015.02.001 25797650 10.1016/j.clinph.2015.02.001 PMC6350257

[39] Hallett M. Transcranial Magnetic Stimulation: A Primer. Neuron. 2007;55: 187–199. doi: 10.1016/j.neuron.2007.06.026 17640522 10.1016/j.neuron.2007.06.026

[40] Carson RG, Nelson BD, Buick AR, Carroll TJ, Kennedy NC, Mac Cann R. Characterizing Changes in the Excitability of Corticospinal Projections to Proximal Muscles of the Upper Limb. Brain Stimul. 2013;6: 760–768. doi: 10.1016/j.brs.2013.01.016 23474090 10.1016/j.brs.2013.01.016

[41] Liu H, Au-Yeung SSY. Reliability of transcranial magnetic stimulation induced corticomotor excitability measurements for a hand muscle in healthy and chronic stroke subjects. J Neurol Sci. 2014;341: 105–109. doi: 10.1016/j.jns.2014.04.012 24792099 10.1016/j.jns.2014.04.012

[42] Kobayashi M, Pascual-Leone A. Transcranial magnetic stimulation in neurology. Lancet Neurol. 2003;2: 145–156. doi: 10.1016/S1474-4422(03)00321-1 12849236 10.1016/s1474-4422(03)00321-1

[43] Walter SD, Eliasziw M, Donner A. Sample size and optimal designs for reliability studies. Stat Med. Wiley Subscription Services, Inc., A Wiley Company; 1998;17: 101–110. doi: 10.1002/(SICI)1097-0258(19980115)17:1<101::AID-SIM727>3.0.CO;2-E 9463853 10.1002/(sici)1097-0258(19980115)17:1<101::aid-sim727>3.0.co;2-e

[44] Breusch TS, Pagan AR. A Simple Test for Heteroscedasticity and Random Coefficient Variation. Econometrica. 1979;47: 1287 doi: 10.2307/1911963

[45] Weir JP. Quantifying test-retest reliability using the intraclass correlation coefficient and the sem. J Strength Cond Res. 2005;19: 231–240. doi: 10.1519/15184.1 15705040 10.1519/15184.1

[46] Flansbjer U-B, Holmbäck AM, Downham D, Patten C, Lexell J. Reliability of gait performance tests in men and women with hemiparesis after stroke. J Rehabil Med. 2005;37: 75–82. doi: 10.1080/16501970410017215 15788341 10.1080/16501970410017215

[47] Troni W, Melillo F, Bertolotto A, Malucchi S, Capobianco M, Sperli F, et al Normative Values for Intertrial Variability of Motor Responses to Nerve Root and Transcranial Stimulation: A Condition for Follow-Up Studies in Individual Subjects. Nógrádi A, editor. PLoS One. 2016;11: e0155268 doi: 10.1371/journal.pone.0155268 27182973 10.1371/journal.pone.0155268 PMC4868303

[48] Day BL, Rothwell JC, Thompson PD, Dick JPR, Cowan JM a., Berardelli A, et al Motor Cortex Stimulation in Intact Man. Brain. 1987;110: 1191–1209. doi: 10.1093/brain/110.5.1191 3676698 10.1093/brain/110.5.1191

[49] Wilson SA, Lockwood RJ, Thickbroom GW, Mastaglia FL. The muscle silent period following transcranial magnetic cortical stimulation. 1993;114: 216–222. 8445404 10.1016/0022-510x(93)90301-e

[50] Jung NH, Delvendahl I, Kuhnke NG, Hauschke D, Stolle S, Mall V. Navigated transcranial magnetic stimulation does not decrease the variability of motor-evoked potentials. Brain Stimul. 2010;3: 87–94. doi: 10.1016/j.brs.2009.10.003 20633437 10.1016/j.brs.2009.10.003

